# TRPV1 Blocker HCRG21 Suppresses TNF-α Production and Prevents the Development of Edema and Hypersensitivity in Carrageenan-Induced Acute Local Inflammation

**DOI:** 10.3390/biomedicines9070716

**Published:** 2021-06-23

**Authors:** Oksana Sintsova, Irina Gladkikh, Anna Klimovich, Yulia Palikova, Viktor Palikov, Olga Styshova, Margarita Monastyrnaya, Igor Dyachenko, Sergey Kozlov, Elena Leychenko

**Affiliations:** 1 G.B. Elyakov Pacific Institute of Bioorganic Chemistry, Far Eastern Branch, Russian Academy of Sciences, 159, Pr. 100 let Vladivostoku, 690022 Vladivostok, Russia; irinagladkikh@gmail.com (I.G.); annaklim_1991@mail.ru (A.K.); styshovaolga84@gmail.com (O.S.); rita1950@mail.ru (M.M.); leychenko@gmail.com (E.L.); 2 Branch of the Shemyakin-Ovchinnikov Institute of Bioorganic Chemistry, Russian Academy of Sciences, Prospekt Nauki, 6, 142290 Pushchino, Russia; yuliyapalikova@bibch.ru (Y.P.); vpalikov@bibch.ru (V.P.); dyachenko@bibch.ru (I.D.); 3 Shemyakin-Ovchinnikov Institute of Bioorganic Chemistry, Russian Academy of Sciences, ul. Miklukho-Maklaya 16/10, 117997 Moscow, Russia; serg@ibch.ru

**Keywords:** TRPV1 inhibition, TNF-α, Kunitz, carrageenan, edema, hypersensitivity, inflammation, analgesia, cytokines

## Abstract

Currently the TRPV1 (transient receptor potential vanilloid type 1) channel is considered to be one of the main targets for pro-inflammatory mediators including TNF-α. Similarly, the inhibition of TRPV1 activity in the peripheral nervous system affects pro-inflammatory mediator production and enhances analgesia in total. In this study, the analgesic and anti-inflammatory effects of HCRG21, the first peptide blocker of TRPV1, were demonstrated in a mice model of carrageenan-induced paw edema. HCRG21 in doses of 0.1 and 1 mg/kg inhibited edema formation compared to the control, demonstrated complete edema disappearance in 24 h in a dose of 1 mg/kg, and effectively reduced the productionof TNF-α in both doses examined. ELISA analysis of blood taken 24 h after carrageenan administration showed a dramatic cytokine value decrease to 25 pg/mL by HCRG21 versus 100 pg/mL in the negative control group, which was less than the TNF-α level in the intact group (40 pg/mL). The HCRG21 demonstrated potent analgesic effects on the models of mechanical and thermal hyperalgesia in carrageenan-induced paw edema. The HCRG21 relief effect was comparable to that of indomethacin taken orally in a dose of 5 mg/kg, but was superior to this nonsteroidal anti-inflammatory drug (NSAID) in duration (which lasted 24 h) in the mechanical sensitivity experiment. The results confirm the existence of a close relationship between TRPV1 activity and TNF-α production once again, and prove the superior pharmacological potential of TRPV1 blockers and the HCRG21 peptide in particular.

## 1. Introduction

The treatment of patients with acute or chronic pain is an urgent problem for medicine and a serious challenge for pharmacology. Regardless of the pain type (acute or chronic, peripheral or central, nociceptive or neuropathic), in many cases the initial injury is accompanied by serious inflammation leading to prolonged pain, the inflammation also may be a result of infectious agents or chemical trauma. Today, the positive role of inflammation as a protective and adaptive nonspecific local reaction of the body to the various damaging factors (physical, chemical, biological) has been convincingly proven, and the importance of controlling the inflammatory response has become clear [1].

Many factors are responsible for inflammation development: the activation threshold of so-called pain receptors, the modulation of nociceptive signals during transmission up, neuroplasticity modulation, and central sensitization, so acting on them may result in inflammatory response suppression [2]. Nervous system activity and inflammation closely affect each other. Peripheral innervation disturbance has been demonstrated to lead to flaccid and prolonged inflammation. Therefore, early anti-inflammatory therapy decreases the chance of acute pain becoming chronic [1].

Currently, the nonselective cationic TRPV1 (transient receptor potential vanilloid type 1) channel, also known as the capsaicin selective vanilloid receptor 1, proved to be one of the main targets of proinflammatory mediators [3,4,5]. Activation of TRPV1 in nociceptive neurons triggers the release of neuropeptides and neurotransmitters, which further leads to the generation of action potentials and signal transduction to the central nervous system for pain sensing [6]. Its activation also triggers the peripheral release of pro-inflammatory compounds that can sensitize other neurons to physical, thermal, or chemical stimuli [4]. TRPV1 is expressed by nociceptors in sensory neurons of the vagus and trigeminal nerves; in the sympathetic plexuses of the intestine and urinary bladder; in the central nervous system neurons, especially in the striatum, cerebellar nucleus, and hippocampus; in the dopaminergic neurons of the substantia nigra and hypothalamus; in various layers of the cerebral cortex; and in non-neuronal cells including keratinocytes, cells of the immune system, smooth muscles, liver, and pancreas, as well as the epithelial cells of various tissues [7,8].

Numerous studies have shown that TRPV1 is also involved in the regulation of a number of pathological conditions, such as, for example, esophageal reflux disease, fecal incontinence, allergic contact dermatitis, airway hypersensitivity, cardiovascular disorders, diabetic and peripheral neuropathy, and pain associated with cancer [9]. Due to its diverse functional role in living organisms, TRPV1 channel is important object of structural and biochemical analysis to create a drug for pain treatment and other pathologies relief as well [10]. Traditional analgesics available for pain and inflammation treatment, according to their main cellular target, suppress inflammatory processes via prostaglandin production inhibition or antinociceptive system activation, whereas TRPV1 antagonists may cause longer analgesia by sensory neuron desensitization. Therefore, they should be considered as a viable alternative to traditional analgesics with a large set of side effects.

To date, from natural sources a significant amount of low-molecular-weight compounds [11] and several peptide ligands to TRPV1 have been isolated, but all of them are either activators (capsaicin, resiniferatoxin, VaTx1-3, DkTx, RhTx, BmP01) or partial inhibitors (APHC1-APHC3, AG489) of this channel [12]. Recently, we found the first TRPV1 blocker, a Kunitz-type peptide, HCRG21, produced by the sea anemone *Heteractis crispa*, which inhibited up to 95% of the TRPV1 currents [13]. Moreover, the peptide was effective in the model of thermal pain stimulation and had a long-term analgesic effect [14]. In the present work, we continued to study the analgesic and anti-inflammatory effects of HCRG21 in a model of carrageenan-induced pain and inflammation and showed that the peptide had a powerful analgesic effect accompanied with a dramatic decrease in TNF-α production mediated by TRPV1.

## 2. Materials and Methods

### 2.1. Production of Recombinant Peptide

HCRG21 was produced as described previously [13]. In brief, DNA encoding the peptide was assembled from the synthetic oligonucleotides in two rounds of PCR reaction and cloned into the vector pET32b+ (Merck KGaA, Darmstadt, Germany). The expression construction pET32b(+)/hcrg21 was used for *Escherichia coli* BL21 (DE3) cell transformation by electroporation on a Multiporator (Eppendorf, Hamburg, Germany) device. BL21 (DE3) cells expressing the fusion protein *TRX*-6His-HCRG21 (after 0.2 mM IPTG induction) were cultured at 18 °C for 16–18 h. Fusion protein was purified using a Ni-NTA-agarose (Qiagen, Hilden, Germany). Cleavage of fusion peptide was performed by addition of HCl up to 0.5 M and CNBr with a molar ratio to protein of 600:1 for 18 h at RT in the dark, as described in [15]. The RP-HPLC was carried out on a Jupiter C4 column (10 × 250 mm) (Phenomenex, Torrance, CA, USA). The solvents A and B were 0.1% TFA in water and in acetonitrile, respectively. The chromatographic separation was performed using a 0–70% gradient of solvent B over 70 min at a flow rate of 3 mL/min. UV detection was monitored at 214 nm.

The purity of target peptide was verified by mass spectrometry. The MALDI-TOF MS spectrum was recorded using an Ultra Flex III MALDI-TOF/TOF mass spectrometer (Bruker, Bremen, Germany) with a nitrogen laser (Smart Beam, 355 nm), reflector, and potential LIFT tandem modes of operation. Sinapinic acid was used as a matrix.

The HCRG21 fold correctness was assessed using NMR spectroscopy on a Bruker Avance III 700 MHz spectrometer (Bruker Biospin, San Jose, CA, USA) equipped with a triple resonance z-gradient TXO probe, as described previously [16].

### 2.2. Ethics Statement

All experimental protocols conform fully to the World Health Organization’s International Guiding Principles for Biomedical Research Involving Animals and were approved locally by the Institutional Commission for the Control and Use of Laboratory Animals of the Branch of the Shemyakin-Ovchinnikov Institute of Bioorganic Chemistry of the Russian Academy of Sciences (protocol number: 700/19, date of approval: 7 June 2019) and the G.B. Elyakov Pacific Institute of Bioorganic Chemistry, Far Eastern Branch, Russian Academy of Sciences (protocol number: 2/20, date of approval: 18 September 2020).

### 2.3. Animals

Adult male ICR mice (Animal Breeding Facility Branch of Shemyakin-Ovchinnikov Institute of Bioorganic Chemistry, Russian Academy of Sciences, Pushchino, Russia, and Animal Breeding Facility G.B. Elyakov Pacific Institute of Bioorganic Chemistry, Far Eastern Branch, Russian Academy of Sciences, Vladivostok, Russia) weighing 20–25 g were used. The animals acclimated in the laboratory for five days. They were kept at room temperature (23 ± 2 °C) with a 12 h light–dark cycle; food and water were available ad libitum. Experimental animals were randomly divided into groups of seven individuals. A dry HCRG21 sample was dissolved in sterile saline and administered intravenously in doses of 0.1 and 1 mg/kg. Negative control animals received an equivalent volume of sterile saline intravenously. Positive control animals received indomethacin (Sigma Aldrich, St. Louis, MO, USA) orally in a dose of 5 mg/kg. Each animal received 20 μL of a 1% solution of delta-carrageenan (Sigma Aldrich, St. Louis, MO, USA) in the hind paw pad after 30 min of saline or HCRG21 treatment and after 60 min of indomethacin treatment due to different administration routes. Intact mice were administrated saline intravenously as well as via the hind paw pad in a volume of 20 μL per animal to induce injection damage. All experiments were carried out as a double-blinded analysis.

### 2.4. Acute Toxicity of HCRG21

Peptide HCRG21 was administered intravenously in doses of 0.1, 1, and 5 mg/kg, and the control group received saline. Changes in basic physiological parameters, such as motility, behavioral responses, and physical activity, were observed in each group for 24 h.

### 2.5. Open-Field Test

Open-field locomotor activity was measured for non-treated animals by a system counting interruptions using a set of photo beams (OPTO—VARIMEX (Columbus Instruments, Columbus, OH, USA) and ATM3 Auto System using Auto-Track Version 4.2 software). Spontaneous locomotor activity was recorded for 3 min.

### 2.6. Paw Edema Test

Acute local inflammation was induced by subplantar injection of 1% delta-carrageenan dissolved in PBS. To determine the size of the inflamed edema, the volume of the area of the animal’s paws from the toes to the hock was measured using a plethysmometer (Ugo Basile, Gemonio VA, Italy) before and 1, 2, 3, 4, and 24 h after the injection of carrageenan.

### 2.7. Pincher-Based Algometer Test

Mechanical nociceptive stimulation was provided by a pincher-based algometer (BIORP-1, Bioseb, Vitrolles, France). Mice were gently restrained with a towel on the bench. Algometer forceps were placed around the hind paw, and increasing pressure was applied. Paw withdrawal response to the applied pressure was registered as a pain threshold to assess mechanical hyperalgesia. Measurements were carried out 2, 4, and 24 h after the injection of carrageenan. Data were averaged from three trials with 1 min inter-application intervals.

### 2.8. Hot Plate Test

Thermal hyperalgesia was tested on a Hot-Plate Analgesia Meter (Columbus Instruments, Columbus, OH, USA) set at 53 °C. The animals were placed individually on the preheated hot-plate surface and exposed to heat until nociceptive reaction was registered. The test was discontinued if withdrawal response was not observed for 30 s. The pain threshold was detected as latency to hind paw withdrawal or licking. Measurements were carried out 2, 4, and 24 h after the injection of carrageenan.

### 2.9. Animals Euthanasia Procedure and Blood Sampling

This procedure was conducted as we described in a previous article [16]. The animals were terminally anaesthetized with sodium pentobarbital (40 mg per mouse i.p., Euthatal, Merial Animal Health, Essex, UK) 24 h after carrageenan injection. Then the thoracic cavity was opened and blood was collected in tubes with the ethylenediaminetetraacetic acid (EDTA) directly from the right atrium of the heart (only after acute local inflammation testing). The entire quantity of blood was centrifuged at 2.5 × 10^3^ g for 10 min to remove cells; the blood serum was then aliquoted and stored at −20 °C.

### 2.10. Measurement of TNF-α

Analysis of the blood samples for TNF-α level was conducted using the ELISA diagnostic kit according to the manufacturer’s protocol (CUSABIO BIOTECH Co., Ltd., Houston, TX, USA).

### 2.11. Statistical Analysis

All data are expressed as mean ± S.E. Student’s *t*-test was performed to determine statistical significance.

## 3. Results

### 3.1. Recombinant Peptide Production

The HCRG21 peptide (P0DL86) was obtained according to the scheme described previously [13]. The yield of the recombinant HCRG21 was 8.2 mg/L cell culture (OD A600 = 0.8). The measured molecular weight of peptide was 6228.5 Da, which corresponded to the calculated data and earlier published results [13,17]. According to the ^1^H NMR spectrum the peptide had a well-defined fold.

### 3.2. Acute Toxicity and Open-Field Locomotor Activity

**Figure 1:**
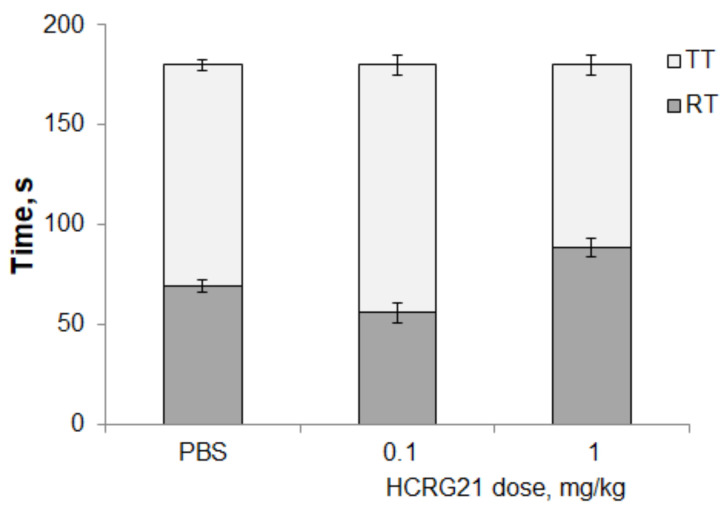
Influence of HCRG21 in doses of 0.1–1 mg/kg on normal mice behavior in the open field test (*n* = 9 for each group). Control animals received a similar volume of PBS. TT—traveling time is the animal movement time; RT—resting time is the animal movement absence time. The results are presented as mean ± S.E.

Intravenous administration of HCRG21 did not cause any toxic effect in doses of 0.1, 1, or 5 mg/kg. According to the results of the open field test, the possible negative effects of HCRG21 on the central nervous system were also excluded. The peptide in doses of 0.1–1 mg/kg did not disturb the normal behavior of animals, and did not cause external intoxication symptoms (convulsions, asphyxia) or mortality. The traveled distance and rearing in the locomotor test were identical to those of the control group treated with saline and the resting-to-traveling time ratio did not alter significantly between the groups (Figure 1). Thus, the efficacy of HCRG21 in in vivo models did not result in locomotor impairment or sedation.

### 3.3. Acute Local Inflammation and Blood TNF-α Level

**Figure 2:**
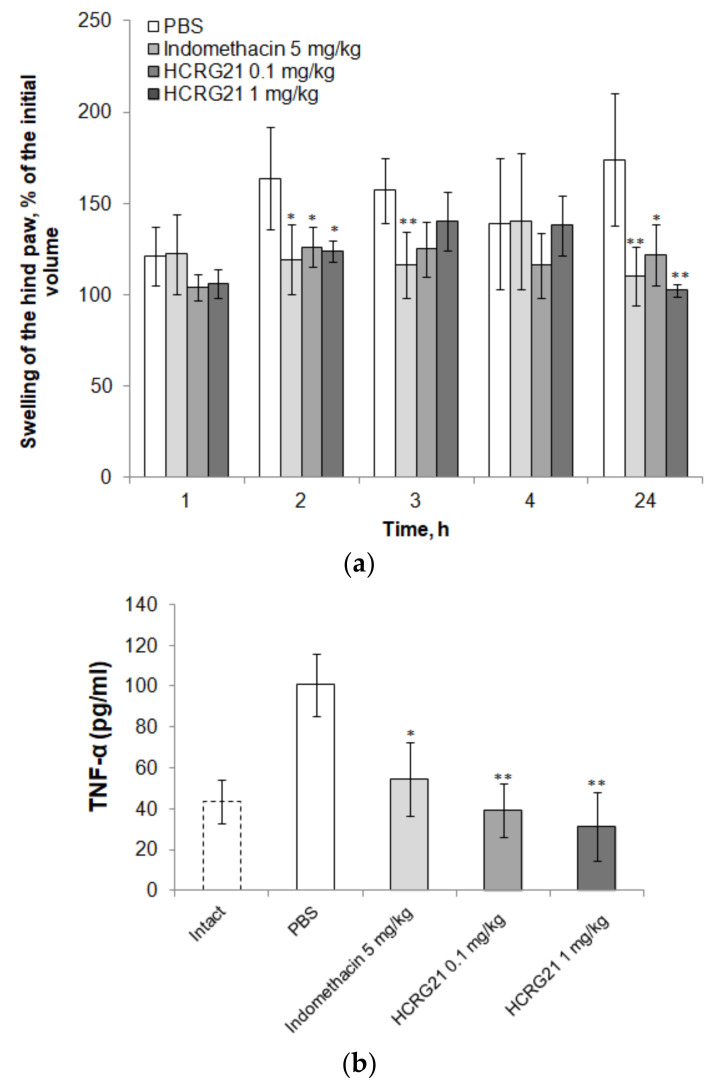
Effect of HCRG21 on (**a**) paw edema and (**b**) TNF-α production (24 h after carrageenan administration). Negative control animals received a similar volume of saline. Positive control animals received indomethacin solution in a dose of 5 mg/kg. Intact animals (**b**) were not subjected to any manipulation. The reliability of differences was calculated by Student’s *t*-test (*n* = 7 for each group). The values * *p* < 0.05, ** *p* < 0.01 are considered reliable in comparison with negative control (PBS).

As a result of the carrageenan administration into the mouse hind paw, edema and redness development were observed in the negative control group (PBS). The local inflammation symptoms were less pronounced in the groups receiving indomethacin and HCRG21. Edema formation inhibition was observed under the action of indomethacin (5 mg/kg) and HCRG21 in doses of 0.1 and 1 mg/kg (Figure 2a). The most noticeable and reliable effect was manifested after 2 and 24 h. HCRG21 in a dose of 1 mg/kg was the most effective, and 24 h after it significantly demonstrated the complete absence of edema, whereas edema in the control group reached the maximum (an increase in paw volume by 70%).

TNF-α is believed to play a leading role in the development of edema and hyperalgesia in this model [18,19]. Indeed, according to the results of an ELISA analysis of blood taken from the negative control animals, the level of TNF-α was 2.4 times higher than in the intact group (Figure 2b). Indomethacin and HCRG21 in both doses effectively reduced the production of TNF-α, and the effect of HCRG21 was dose-dependent. It is noteworthy that HCRG21 reduced the TNF-α level below the values of the intact group. This was especially noticeable in the dose of 1 mg/kg, where the cytokine value decreased up to 25 pg/mL versus 40 pg/mL in the intact group. In this regard, we analyzed the HCRG21 analgesic effect on mechanical and thermal hyperalgesia (also known to be affected by TRPV1 activity) for carrageenan-induced inflammation.

### 3.4. Mechanical Hyperalgesia

**Figure 3:**
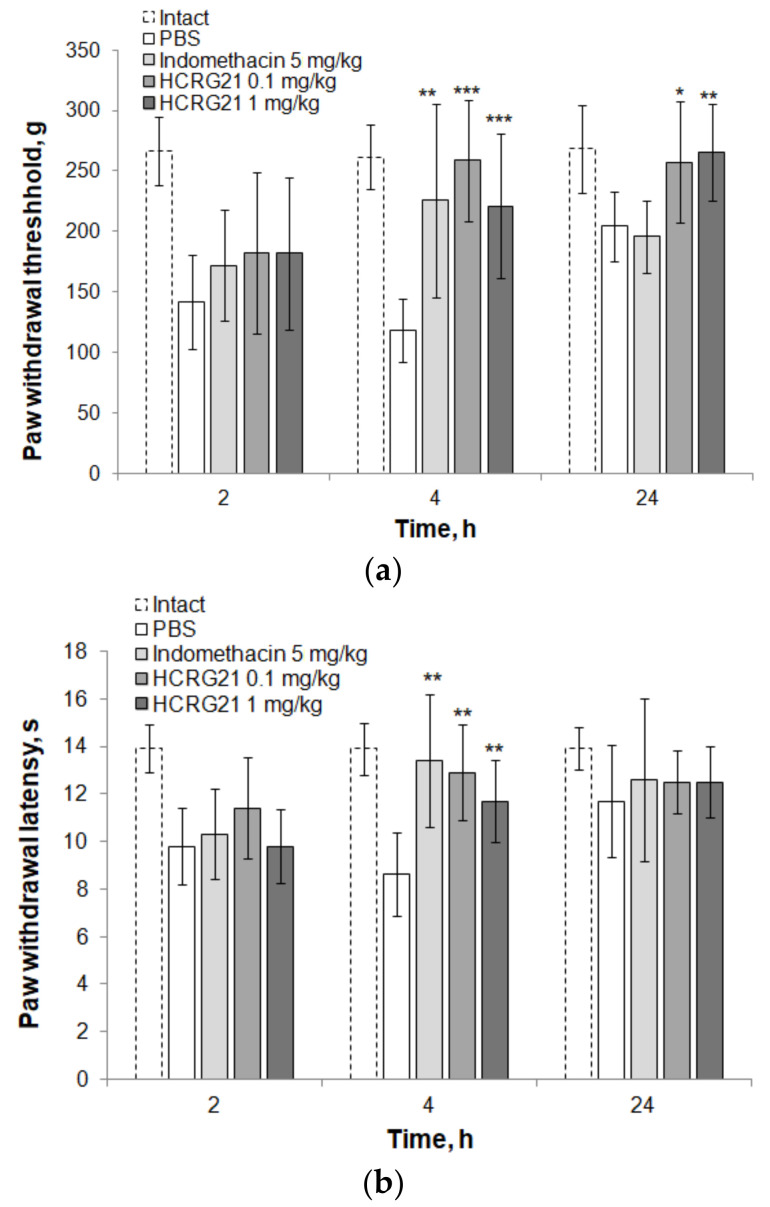
Effect of HCRG21 on mechanical (**a**) and thermal (**b**) hyperalgesia in mice with carrageenan-induced paw edema. Control animals received a similar volume of saline (negative control) or indomethacin solution in a dose of 5 mg/kg (positive control). Intact animals did not have inflammation. The reliability of differences was calculated by Student’s *t*-test (*n* = 7 for each group). The values * *p* < 0.05, ** *p* < 0.01, *** *p* < 0.001 are considered reliable in comparison to the saline group.

Mechanical hyperalgesia during carrageenan-induced paw edema was tested by Pincher-Based Algometer. In the negative control group, the pain threshold was reduced to the maximal sensitivity in 4 h after carrageenan administration (Figure 3a). In contrast, indomethacin and HCRG21 (in both doses) showed a stable increase in the pain threshold during the observation time and significant and effective analgesic effects after 4 and 24 (only HCRG21) hours of observation. The short indomethacin effect and over 24 h analgesia for HCRG21 could be explained by HCRG21 potency to prevent the development of edema and pain response (24 h after, the mice that received the peptide did not differ from the intact ones).

### 3.5. Thermal Hyperalgesia

Thermal hyperalgesia during carrageenan-induced paw edema was studied by hot-plate test. In the negative control group, the pain threshold of thermal sensitivity was lowest 4 h after the carrageenan administration (Figure 3b). Indomethacin and HCRG21 showed significant and effective analgesic effects at the fourth hour of the experiment. The thermal hypersensitivity of the negative control group was comparable to the treated groups 24th hour after carrageenan administration.

## 4. Discussion

Interest in studying the interaction between the immune and nervous systems is growing steadily. To generate pain, a signal from the periphery passes through specialized primary sensory neurons (nociceptors), which can respond to various dangerous thermal, mechanical, and chemical stimuli and transmit information about the danger to the brain [20]. Surrounded by numerous cells, nociceptors are influenced by cytokines, including those responsible for the formation and prolongation of the inflammatory response. Cytokines can cause sensitization of nociceptors both indirectly through signal transduction and directly through their specific receptors presented on the membranes of neuronal cells. In turn, the long-term activation of nociceptors leads to an increase in the level of cytokines in the blood [21]. The search for compounds that inhibit nociceptors and, at the same time, reduce the production of proinflammatory cytokines, seems to be relevant, since they can relieve pain more effectively in comparison to traditional drugs.

This problem can be solved by using peptide HCRG21 as an analgesic agent, which has a well-defined molecular target—the TRPV1 ion channel. It is the first peptide blocker of this channel (inhibits up to 95% of the currents, IC_50_ 6.9 μM) [13] and has a prolonged analgesic effect (up to 13 h) in the model of thermal pain stimulation [14]. We previously demonstrated that HCRG21 was able to suppress the synthesis of reactive oxygen species in nerve cells and increased their survival in a model of 6-hydroxydopamine-induced neurotoxicity that imitates in vitro Parkinson’s disease tightly associated with neuroinflammation [17]. In this study, the potential of HCRG21 was revealed as an effective anti-inflammatory compound. For this purpose, the murine model of carrageenan-induced local inflammation was used. The chosen model reproduced acute local inflammation, characterized by five classic signs: hypersensitivity, local temperature rise, edema, redness, and loss of function. This model made it possible to evaluate the production of inflammatory mediators and the anti-inflammatory and analgesic effects of various substances [18].

Previously, the effectiveness of the peptide in the thermal pain stimulation test was demonstrated in doses of 0.1–1 mg/kg when administered intramuscularly [14]. In this study, HCRG21 demonstrated a significant anti-inflammatory effect in doses of 0.1 and 1 mg/kg when administered intravenously. The peptide’s effect was expressed in suppressing the development of edema and was comparable to NSAID indomethacin effectiveness in a dose of 5 mg/kg. An anti-inflammatory effect in this model was shown for LASSBio-1135 (imidazo[1,2-a]pyridine derivative), a dual inhibitor of TRPV1 and COX-2. Oral administration of 100 µM/kg of LASSBio-1135 markedly reduced thermal hyperalgesia induced by carrageenan, but at 10 µM/kg only a partial reduction was observed after the fourth hour. Moreover, TNF-α production after carrageenan stimulus was also inhibited by treatment with LASSBio-1135 at 10–100 µM/kg [22]. Correlation between the inhibition of TRPV1 currents and the production of TNF-α was demonstrated for neuroprotectin-1 (NPD1), which is an anti-inflammatory endogenous lipid mediator. NPD1, an indirect TRPV1 current inhibitor, blocked TNF-α-mediated synaptic transmission of the spinal cord. Spinal injection of NPD1 at doses of 0.1–10 ng/kg inhibited TRPV1- and TNF-α-dependent inflammatory pain [23]. There is also evidence of TNF-α’s ability to increase the sensitivity of TRPV1 up to spontaneous current development, thereby causing pain [24,25,26].

In our study, we observed a sharp increase in the level of TNF-α for the negative control group treated with carrageenan compared to the intact group, which was consistent with the data of previous experiments [18]. TNF-α is a potent pro-inflammatory cytokine that has many effects, including the activation of inflammatory cells, induction of several inflammatory proteins, cytotoxicity, apoptosis, etc. [21]. TNF-α showed a leading contribution to the development of inflammatory (edema and neutrophil migration) and nociceptive responses induced by carrageenan in two models: pleural inflammation and carrageenan-induced paw edema [18,19]. Nociceptive response was significantly reduced in TNF-α-p55-receptor knockout mice, as well as in mice systemically receiving anti-TNF-α antibody. Inflammatory and nociceptive responses were also decreased when mice were previously treated with the preferential inhibitor of TNF-α production, thalidomide [18].

The treatment of mice with indomethacin and HCRG21 significantly reduced TNF-α production compared to PBS, and animals showed approximately equal results in tests, but HCRG21 had a more prolonged effect in the mechanosensitivity test (Figure 3a), which can be explained by the action on different molecular targets. Indomethacin inhibition of COXs leads to suppression of the lipid-derived inflammatory mediators (prostaglandin E2 (PGE2), prostacyclin (PGI2), prostaglandin D2 (PGD2), and prostaglandin F2α (PGF2α)) production [27]. The initial phase of carrageenan edema (0–1 h), which is not inhibited by NSAIDs, is attributed to the release of histamine, 5-hydroxytryptamine, and bradykinin, followed by a late phase (1–6 h) sustained mainly by prostaglandin release and attributed to the induction of inducible COX-2 in the tissue [28]. In our experiment, we observed a similar pattern of edema development (Figure 2a). The edema formed in two control animal groups, treated with indomethacin and PBS, 1 h after carrageenan administration, but after the second hour, indomethacin decreased the edema formation. In contrast, HCRG21 began to inhibit the development of edema immediately after carrageenan administration. The action of HCRG21 at the initial phase of inflammation may be a result of the TRPV1 involving in the transmission of signals from histamine receptors (H1R and H4R) into the CNS [29,30,31].

Our results of TNF-α level reduction were shown for the first time for a peptide inhibitor of TRPV1. In addition, we found that the level of TNF-α production was lower than in intact animals, which was previously shown for LASSBio-1135, which strongly reduced TNF-α production during inflammation to the level of saline-treated animals [22]. Taken together, these experimental data suggest the presence of both mechanisms of inhibition of carrageenan-induced edema and hyperalgesia—a reduction in TNF-α production in immune cells and a reduction in neuronal activity by antagonizing TRPV1—and once again confirm the existence of a close relationship between TRPV1 and TNF-α.

HCRG21 had superior effectiveness over indomethacin at 24 h in mechanical hyperalgesia (Figure 3a), which suggests a long anti-inflammatory effect of the peptide in addition to the prolonged analgesic effect measured [14]. HCRG21 did not cause signs of toxicity in doses of up to 5 mg/kg. In addition, its close analogue, APHC3, was also non-toxic, even within a course of 14 days in high doses (up to 100 mg/kg) [32]. As a result, HCRG21 is a promising molecule for the suppression of pain and inflammation, including emergency interventions—for example, inflammation from acute infections such as COVID-19. The significant pharmacological potential of TRPV1 blockers and the HCRG21 peptide in particular for the rapid and effective suppression of inflammation was proven in this study and gives hope for the expansion of the range of analgesic drugs in the future.
